# Intestinal Protozoa, Helminth Infection, and Associated Factors among Tuberculosis Patients and Nontuberculosis Persons in Bobo-Dioulasso City, Burkina Faso

**DOI:** 10.4269/ajtmh.23-0346

**Published:** 2024-09-24

**Authors:** Diakourga Arthur Djibougou, Gloria Ivy Mensah, Mamoudou Cissé, Toé Inoussa, Leon Tinnoga Sawadogo, Adjima Combary, Adama Sanou, Bassirou Bonfoh, Kennedy Kwasi Addo, Adrien Marie Gaston Belem, Clément Ziemlé Meda, Roch Konbobr Dabiré, Achille Kaboré, Potiandi Serge Diagbouga

**Affiliations:** ^1^ Université Nazi BONI, Bobo-Dioulasso, Burkina Faso;; ^2^ Centre MURAZ/Institut National de Santé Publique, Bobo-Dioulasso, Burkina Faso;; ^3^ Institut de Recherche en Sciences de la Santé/Centre National de Recherche Scientifique et Technologique, Ouagadougou, Burkina Faso;; ^4^ Department of Bacteriology, Noguchi Memorial Institute for Medical Research, College of Health Sciences, University of Ghana, Legon, Accra, Ghana;; ^5^ Programme National Tuberculose, Ministry of Health, Ouagadougou, Burkina Faso;; ^6^ Centre Suisse de Recherches Scientifique de Côte d’Ivoire, Abidjan, Côte d’Ivoire;; ^7^ Family Health International 360 (FHI 360), Washington, District of Columbia;; ^8^ Etudes Formation et Recherches Développement en Santé (EFORDS), Ouagadougou, Burkina Faso

## Abstract

We report the frequency and associated factors of tuberculosis (TB) and parasite coinfection from newly diagnosed pulmonary TB patients (TB+) and non-TB participants (TB−) from the Regional Tuberculosis Control Center, households, and health facilities in Bobo-Dioulasso from 2019 to 2021. Biological samples were examined for parasite infection using direct microscopy, concentration techniques, and the immunochromatographic rapid test. Data were analyzed using STATA 14. Of a total of 192 participants involved, 95 were TB+ and 97 were non-TB. There was no statistically significant difference in parasitic infections between the two groups, although it was higher in TB+ than TB− (69.5% [66/95] versus 55.7% [54/97]; *P* = 0.07). Protozoal infection prevalence was significantly higher in patients with TB+ than in those TB− (61.1% versus 37.1%; *P* = 0.001). Specifically, *Entamoeba* spp. and *Cryptosporidium* spp. followed this pattern with 35.8% versus 19.6% (*P* = 0.01) and 22.1% versus 8.3% (*P* = 0.007), respectively. Although higher in TB+ patients, helminthiasis frequency was not significantly different between the two groups (23.2% versus 15.5%; *P* = 0.2). Helminth species were *Schistosomia mansoni* (17.9% versus 12.4%), *Dicrocoelium dendriticum* (3.2% versus 1.0%), *Enterobius vermicularis* (2.1% versus 2.1%), *Wuchereria bancrofti* (1.1% versus 0.0%), and *Hymenolepis nana* (1.1% versus 0.0%). Illiteracy (adjusted odds ratio [aOR]: 2.5; 95% CI: 1.0–6.1), smoking (aOR: 2.4; 95% CI: 1.1–5.3), and handwashing after defecation (aOR: 2.4; 95% CI: 1.2–4.7) were associated with parasites. This study reported a high frequency of parasite coinfection in TB patients. These findings suggest the need for adequate health education for behavioral change and systematic diagnosing of parasites in TB patients for better coinfection management.

## INTRODUCTION

Tuberculosis (TB) is an infectious disease caused by mycobacteria of the *Mycobacterium tuberculosis* complex (MTBC) and is a major global health problem. Indeed, it is estimated that more than 2 billion people are infected with *M. tuberculosis* (*Mtb*) worldwide.^1^ It has caused 10.1 million new cases, including 1.2 million deaths (WHO, 2020). In 2020, most TB cases were in Southeast Asia (43%) and Africa (25%).^2^

In addition, at least 3.5 billion people are infected with foodborne geo-helminths, schistosomes, filarial worms and trematodes, and intestinal protozoa,^3^ which are also a public health problem despite control initiatives such as mass administration of albendazole and ivermectin. In sub-Saharan Africa, these parasitic infections rates are very high at 37–48%.^4^

Tuberculosis and parasitic infections share similar geographical contexts, particularly in tropical and subtropical regions,^5^ where 20–35% of people with TB are infected with helminths.^6^ Several factors contribute to the occurrence of the TB and parasites pair, including poor living conditions, lack of food and body hygiene, the biotope of the populations, malnutrition, overpopulation, lack of deworming, the emergence of drug-resistant strains, and the level of education or ignorance of the modes of transmission.^7^^,^^8^

Parasitic infections, particularly helminthiasis, affect the host and lead to alterations in the immune response, favoring the progression of infection by MTBC agents.^9^ Indeed, helminths induce immunomodulatory responses that allow them to persist in the host for years, with a humoral T helper 2 (Th2) immune response and downregulation of T helper 1 (Th1) (most notably interferon-γ and cytotoxic T lymphocyte) responses.^10^^,^^11^ In fact, dendritic cells (DCs) are well-known to play an essential role in presenting antigens to T cells to initiate immune responses. However, the function of these DCs is altered by helminths, something that clearly promotes both Th2 and regulatory responses.^12^ This alteration of the host immunological milieu contributes to an altered immunological response to the *Mtb* complex, which requires Th1 responses to limit the severity and progression of the infection.^11^

Owing to the susceptibility of HIV patients to TB, sub-Saharan Africa has been disproportionately affected and accounts for four of every five cases of HIV-associated TB. Immunocompromised diseases such as HIV/AIDS are associated with TB. Indeed, in concurrent HIV and *Mtb* infection the interleukin-4/tumor necrosis factor-α interaction may be exacerbated. The depletion of T cells makes the host vulnerable to HIV progression, increases the risk of progression to active TB, and reactivates latent TB infection.^13^

Tuberculosis and parasites coinfection has been reported in different countries around the world,^3^^,^^10^^,^^14^^–^^16^ and it was revealed that the prevalence of parasitic infections was higher in TB patients than in those without TB.^8^^,^^17^^,^^18^ In Brazil, it was found that 57.8% of TB patients had an intestinal parasite.^16^ Parasite infestation was found in 14.9% of pulmonary TB patients in China^19^ and 70.9% in Ethiopia.^5^

Studies on coinfection between TB and parasitic infestations are very rare in West Africa, more specifically in Burkina Faso. However, despite mass drug distribution campaigns, parasites have always persisted.^20^^–^^24^

Understanding the frequency of the phenomenon would help develop an effective prevention and control strategy to reduce mortality related to TB and parasitic diseases coinfection. In this study, we evaluated the frequency and factors associated with parasitic coinfection in patients with active pulmonary TB in Bobo-Dioulasso in western Burkina Faso.

## MATERIALS AND METHODS

### Study site and period.

**Figure 1.**
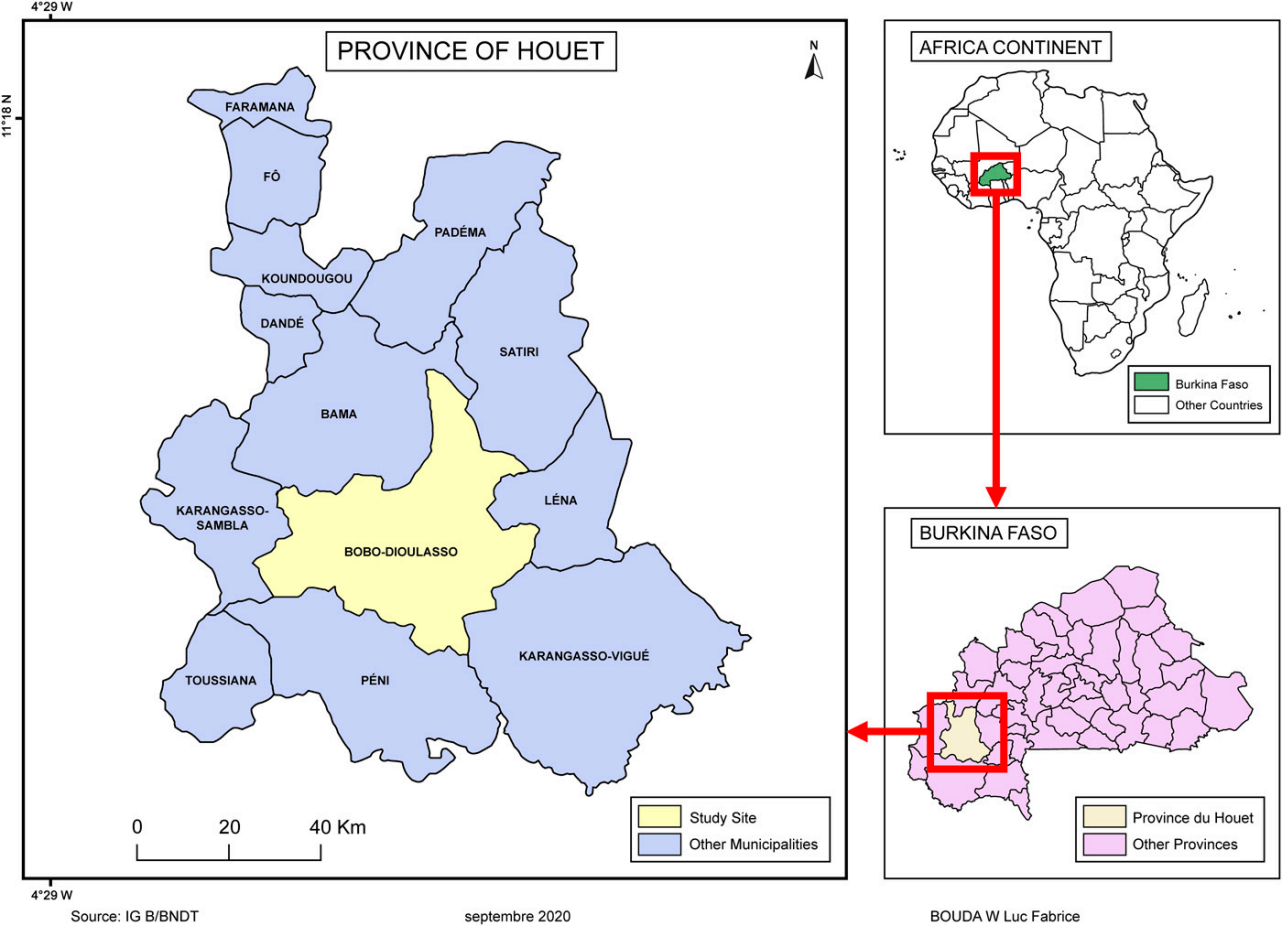
Map of the study area.

We conducted a comparative cross-sectional study from March 2019 to July 2021 in Bobo-Dioulasso (11°10′42′′N; 4°17′35′′W); the economic capital is in the western part of the country. The overall prevalence of opportunistic intestinal parasitic infections at the reference hospital in Bobo-Dioulasso was 65.3% in 2015,^25^ and the incidence of TB was estimated at 45/100,000 inhabitants in 2021.^26^ The collection sites for the study were the regional TB control center, the Dafra medical center, the Do medical center, and the Bobo-Dioulasso abattoir (Figure 1).

### Population, questionnaire administration, and sample collection.

The study participants consisted of TB patients (TB+) recruited from the Regional Tuberculosis Control Center and non-TB participants (TB−), who consisted of contact persons of TB index cases, human and animal health workers recruited from households, health facilities, and slaughterhouse. Tuberculosis patients were newly diagnosed pulmonary TB patients with molecular amplification of *Mtb* complex DNA in their sputum obtained with the GeneXpert MTB/RIF technique (Cepheid, Sunnyvale, CA) and who gave informed consent. Non-TB participants (TB−) were contact persons of TB index cases and human and animal health workers (free of symptoms and clinical signs suggestive of TB) who provided informed consent. They were chosen to be a mixed healthy control group. A questionnaire was used to collect demographic data such as age, sex, occupation, educational level, smoking status, clinical data, and presence of animals in the household or compound such as goats, sheep, cows, pigs, donkeys, cats, and poultry. Before the questionnaire was administered, recruited participants who had completed informed consent forms were assigned unique identification numbers. Each participant’s unique identification number was used to allocate a pre-labeled sterile stool specimen bottle and urine bottles as well as blood tubes. Participants who tested positive for TB or parasite infection provided their samples before receiving their anti-TB or parasitic infestation treatment.

### Laboratory procedures.

We collected one stool sample, one urine sample (for parasitological analysis), and one 4-mL blood sample in an ethylenediaminetetraacetic acid tube (for immunohematology testing) from each participant. All samples were analyzed at the Laboratory of Parasitology of Center MURAZ, together with completed collection sheets.

#### Parasitological analysis.

##### Analyses of stool samples.

For the formalin-ether and Kato-Katz concentration techniques, stool samples were processed using the formalin-ether concentration method described previously.^27^ The concentration pellets were placed on a slide and examined using 10× and 40× objectives. A portion of each stool sample was subjected to the Kato-Katz technique (single thick smears, using a standard 41.7-mg template) as described elsewhere.^28^ The processed specimen was sampled for one slide and examined by two parasitologists using 10× and 40× objectives.

For the search for *Cryptosporidium* spp. by modified Ziehl-Neelsen (MZN)^28^ stain, smears were made from the pellets obtained by the formalin-ether concentration method. The MZN-stained smears were examined under a microscope at 1,000× objective for *Cryptosporidium* spp. oocysts, which appeared pink to red, surrounded by a clear halo on a blue background.

##### Processing of urine samples.

Urine samples were examined qualitatively for *Schistosoma* spp. using the urine sedimentation method and rapid circulating cathodic antigen point-of-care test (CCA-POC), as described previously.^22^

##### Serological diagnosis of lymphatic filariasis and HIV.

Testing for anti-lymphatic filariasis IgM and IgG was performed using the Filariasis IgG/IgM Combo rapid test (CTK Biotech, Poway, CA) from the serum of study participants following the manufacturer’s procedures.

The HIV testing was performed using the Alere Determine HIV rapid test (Alere; San Diego, CA) and the Onsite HIV 1/2 Ab Plus Combo Rapid test (CTK Biotech) following the HIV testing algorithm in Burkina Faso.

## STATISTICAL ANALYSES

Data collected from the questionnaires and the results of the tests performed were entered into Microsoft Excel 2016 spreadsheet software and then exported to STATA 14 (STATA Corp., College Station, TX) for statistical analyses.

Descriptive statistics were performed by determining the proportions for categorical variables and the mean or median for continuous variables. In addition, χ^2^ and Fisher’s exact tests were used to compare proportions. These comparisons were made for protozoan and helminth infections between the study arms (TB+ and TB− groups).

In addition, univariate and multivariable logistic regression models were used to assess the association between parasitic infection status and potential predictors. To do this, we first performed univariate logistic regression, and only variables with a *P*-value ≤0.10 were included in the multivariate regression. The variables included in the multivariate logistic regression were type of participant (TB+ or TB−), sex, occupation, body mass index (BMI), educational level, smoking, handwashing, deworming, and coughing. A *P*-value <0.05 was considered statistically significant.

## RESULTS

### Sociodemographic characteristics of the study population.

**Figure 2.**
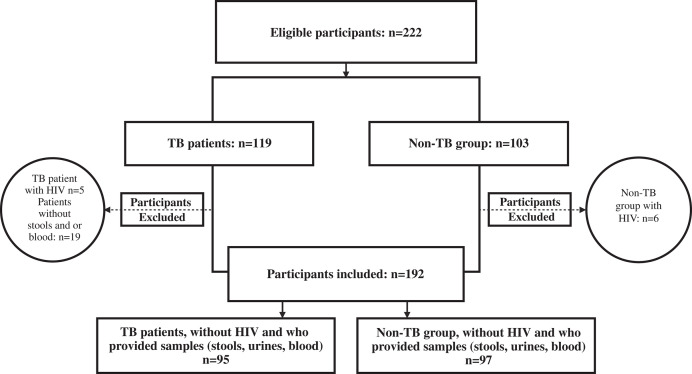
Study flowchart. TB = tuberculosis.

A total of 95 TB+ and 97 TB− patients were recruited for the study out of an initial total of 222 eligible subjects (Figure 2). The mean age of the study participants was 37.7 ±13.0 years, and 76.6% (147/192) of the participants were male. The TB+ patients were more likely to be male than the non-TB group (85.3% versus 68.0%). The TB+ patients also had a mean BMI of 18.0 ± 2.3 kg/m^2^, compared with 24.5 ± 3.8 kg/m^2^ in the non-TB controls (Table 1).

**Table 1:** Sociodemographic characteristics of TB patients and controls

Variables	Category	Patients with TB	Control Group	Total
		*n* (%)	*n* (%)	
Sex	Female	14 (14.7)	31 (32.0)	45 (23.4)
	Male	81 (85.3)	66 (68.0)	147 (76.6)
Age	Mean (SD)	37.6 (14.0)	37.9 (12.1)	37.7 (13.0)
BMI, kg/m^2^	Mean (SD)	18.0 (2.3)	24.5 (3.8)	21.3 (4.6)
Level of Education	Illiterate	44 (46.3)	17 (17.5)	61 (31.8)
	Literate	51 (53.7)	80 (82.5)	131 (68.2)
Smoking	No	46 (48.4)	77 (79.4)	123 (64.1)
	Yes	49 (51.6)	20 (20.6)	69 (35.9)
Alcoholism	No	86 (90.5)	66 (68.0)	152 (79.2)
	Yes	9 (9.5)	31 (32.0)	40 (20.8)
Diabetes	No	48 (96.0)	85 (87.6)	133 (90.5)
	Yes	2 (4.0)	12 (12.4)	14 (9.5)
Chronic Cough	No	00 (0.0)	87 (89.7)	87 (45.3)
	Yes	95 (100.0)	10 (10.3)	105 (54.7)
Handwashing before Meal	Occasionally	42 (44.2)	14 (14.4)	56 (29.2)
	Regularly	53 (55.8)	83 (85.6)	136 (70.8)
Handwashing after Defecation	No	36 (37.9)	00 (0.0)	36 (18.8)
	Yes	59 (62.1)	97 (100.0)	156 (81.2)
Toilet Use	No	2 (2.1)	00 (0.0)	2 (1.0)
	Yes	93 (97.9)	97 (100.0)	190 (99.0)
Dirty Water disposal	Channels	5 (5.3)	3 (3.1)	8 (4.2)
	Septic Tanks	22 (23.2)	71 (73.2)	93 (48.4)
	On the Road	68 (71.6)	23 (23.7)	91 (47.4)
Deworming in the Last 6 Months	No	93 (97.9)	58 (59.8)	151 (78.6)
	Yes	2 (2.1)	39 (40.2)	41 (21.4)
Presence of Animals	No	49 (51.6)	44 (45.4)	93 (48.4)
	Yes	46 (48.4)	53 (54.6)	99 (51.6)

BMI = body mass index; TB = tuberculosis.

### Frequency of parasitic infections.

In this study, the overall frequency of protozoan infections was significantly higher in TB cases than in non-TB cases (61.1% versus 37.1%; *P* = 0.001). In particular, the frequency of *Entamoeba* spp. and *Cryptosporidium* spp. followed this pattern with 35.8% versus 19.6%; *P* = 0.01 and 22.1% versus 8.2%; *P* = 0.007, respectively. Helminthic infections were not significantly different between the two groups (23.2 versus 15.5; *P =* 0.2). The helminths found were *Schistosoma mansoni*, *Dicrocoelium dendriticum, Enterobius vermicularis, Wuchereria bancrofti*, and *Hymenolepis nana* in the proportions of 17.9%, 3.2%, 2.1%, 1.1%, and 1.1% respectively, in the TB patients compared with 12.4%, 1.0%, and 2.1% in the non-TB patients, where there were no *W. bancrofti* or *H. nana* infections. Overall, we did not find a statistically significant difference in parasitic infestation, although TB+ patients were more infested than the control group (69.5% versus 55.7%; *P =* 0.07). Polyparasitism was more common than monoparasitism, with proportions of 35.8% versus 33.7% and 24.7% versus 19.6% in TB and non-TB patients, respectively (Table 2).

**Table 2:** Proportion of parasite species infections among patients with TB and non-TB persons

Status	TB Cases	Non-TB Persons	χ^2^	*P*-Value
	(*N* = 95)	(*N* = 97)		
	*n* (%)	*n* (%)		
Protozoa Infections				
* Entamoeba* spp.	34 (35.8)	19 (19.6)	6.3	0.01
* Cryptosporidium* spp.	21 (22.1)	8 (8.3)	7.2	0.007
* Endolimax nana*	15 (15.8)	21 (21.7)	1.1	0.3
* Giardia intestinalis*	7 (7.4)	9 (9.3)	0.2	0.6
* Blastocystis hominis*	2 (2.1)	0 (0.0)	–	0.2*
* Trichomonas intestinalis*	0 (0.0)	1 (1.0)	–	1*
Total Protozoa Infection	58 (61.1)	36 (37.1)	11.0	0.001
Helminth Infections				
* Schistosoma mansoni*	17 (17.9)	12 (12.4)	1.1	0.3
* Dicrocoelium dendriticum*	3 (3.2)	1 (1.0)		0.3*
* Enterobius vermicularis*	2 (2.1)	2 (2.1)	–	1*
* Wuchereria bancrofti* IgM	1 (1.1)	0 (0.0)	–	0.5*
* Hymenolepis nana*	1 (1.1)	0 (0.0)	–	0.5*
Total Helminth Infection	22 (23.2)	15 (15.5)	1.8	0.2
Type of infection				
Polyparasitism	34 (35.8)	24 (24.7)	2.8	0.1
Monoparasitism	32 (33.7)	19 (19.6)	4.9	0.03
Total Parasite Infection	66 (69.5)	54 (55.7)	3.9	0.07

TB = tuberculosis.

* *P*-value for Fisher’s test.

### Factors associated with parasitic infections.

Illiteracy (adjusted odds ratio [aOR]: 2.5; 95% CI: 1.0–6.1), smoking (aOR: 2.4; 95% CI: 1.1–5.3), and handwashing after defecation (aOR: 2.4; 95% CI: 1.2–4.7) were significantly associated with parasitic infections.

## DISCUSSION

This study aimed to assess the frequency of parasitic infections and associated factors in TB patients versus the non-TB group in Burkina Faso. Although it is often ignored, it is worth noting that TB and parasites share similar geographic areas, especially in developing countries.^10^^,^^29^

The rates of parasitic infections in TB patients were high at 62.4%, although there was no statistically significant difference compared with rates in the non-TB group (*P =* 0.07). Similar results were reported in Brazil (57.8%)^16^ and in Iraq 67.3%.^30^ Independently, our results were higher than those reported in China (14.9%),^31^ Iran (21.1%),^32^ Uganda (4.7%),^7^ and different regions of Ethiopia, namely Arba Minch (26.3%)^33^ in Adama town (21.4%)^34^ and in Gondar (33.3%).^14^ Our rate was lower than the 71% reported in TB patients versus 36% in controls in Gondar in 2006.^5^ The disparity in infection rates could be explained by the history of mass drug administration with ivermectin and albendazole or other control programs for parasitic infections and by differences in TB case management. Furthermore, the geographic characteristics of the study sites, which would be either favorable or unfavorable to the life cycle of parasites, the socioeconomic level of the populations, and the study sample sizes as well as the selection criteria of the study participants. The quality or the number of sample sets collected and the methods of stool examination (in our study at least four different techniques were used to search for parasites) should also be considered as a possible reason.

However, in Burkina Faso, recent studies have reported high frequencies of intestinal parasites in school-aged children (81.1%)^22^ and public hospital patients (65.3%),^25^ whereas there is no evidence of TB and parasite coinfection in the country. Our study results raise the need for national TB programs to implement an integrated TB management strategy involving the diagnosis of parasites in new TB patients.

Infections with intestinal protozoa were significantly higher in TB patients than in non-TB participants (61.1% versus 37.1%; *P* = 0.001). High frequencies have been reported in other countries such as Brazil (40%). This high frequency could be explained by the fact that these parasites are mainly transmitted by the fecal-oral, zoonotic, or anthroponotic route through contaminated water or food, and the low level of knowledge or ignorance of the modes of contamination of these parasites, as well as a failure of local strategies to mitigate transmission of the diseases.^35^ The protozoan infection should not be neglected because it was reported in previous studies in Burkina Faso.^36^

Specifically, *Entamoeba* spp. and *Cryptosporidium* spp. infestations were significantly higher in TB patients than in non-TB participants (35.8% versus 19.6%; *P* = 0.001 and 22.1% versus 8.3%; *P* = 0.01, respectively). Other studies have reported similar results for amoebae elsewhere in Ethiopia (40%), in Brazil (75%),^37^ and in Iran (18.6%),^32^ all of which support the hypothesis that amoebae are reservoirs par excellence of mycobacterial species.^38^^–^^40^ Indeed, *Mtb* ingested by amoebae grow intracellularly, acquiring an invasive phenotype when the bacteria escape the infected amoeba. Amoebae can provide a replication niche and serve as a reservoir for bacteria, contributing to the survival of TB complex mycobacteria in soil.^39^ Consequently, in our context, there is a high risk of amoeba infection, especially in areas where there is a lack of or insufficient water, hygiene, and sanitation.

*Cryptosporidium* spp. were reported at 22.1% in our study. Similar results tend to increase in TB patients in other studies, like those in Ethiopia (6.0%),^41^ Mozambique (7.1%),^42^ Iran (1.2%),^32^ Iraq (5%),^30^ and China (1.1%).^31^ This could be attributed to the failure of the immune system in TB patients, thus making them more susceptible to opportunistic infections such as *Cryptosporidium* spp.

The overall frequency of helminthic infections was not significantly different between the two groups (23.2% versus 15.5%; *P =* 0.2). However, the high rate of helminthic infections at 23.2% in TB patients was similar to results reported in Arba Minch, Ethiopia (24.4%),^33^ Tanzania (31.8%),^3^ Brazil (27.5%),^43^ and northwest Ethiopia (29%).^10^ However, our rate was lower than that reported in Ethiopia by Elias et al.^5^ at 71%. In this study, the reported helminthiasis was mainly due to *S. mansoni*, *D. dendriticum*, *W. bancrofti*, *E. vermicularis,* and *H. nana* in TB+ patients, indicating a high diversity of helminths with positive tropism for TB. Indeed, helminth infections induce an opposing anti-inflammatory Th2 and Th3 immune-regulatory response, whereas TB immune response is predominantly proinflammatory Th1 dependent.^44^ Amelio et al.^44^ showed more recently that helminth infection may have caused an induction of mixed Th1/Th2 *Mtb*-specific CD4 T cell responses in TB patients from Tanzania.^44^ The implications for the complex immunological interactions that occur with helminth infections and TB need to be studied in West African settings.

In our study, single-parasitism in TB patients was significantly associated with parasitic coinfection (*P* <0.05) compared with polyparasitism. Similar results have been reported in other studies.^5^^,^^45^ Given the above, raising awareness of personal and environmental hygiene within communities should be a part of the integrated strategy of a control program. This would significantly reduce the prevalence of helminths and mitigate latent reactivation of TB into active TB due to helminth parasites.

Tuberculosis and parasitic infestations, in both cases, could be a risk factor for each other.^5^^,^^46^ In the present study, although TB patients harbored more parasites (protozoa and helminths) than non-TB participants, we did not find an association of TB with parasitic infestations (aOR: 0.7; 95% CI: 0.20–2.7) (Table 3). Similar results have been reported by previous studies conducted in different settings, including the studies conducted in Gondar by Abate et al.^10^ in Ethiopia and by Mhimbira et al.^3^ in Tanzania. However, in other studies, intestinal helminths were found in TB patients with a significant difference from controls, notably in the study by Elias et al.^5^ and Tristão-Sá et al.^16^ This could be due to the epidemiological variability of intestinal helminths across geographic areas.

**Table 3:** Factors associated with parasitic infections in participants by univariate and multivariate regression

Variables	Category	*n* (%)	Parasite “No”	Parasite “Yes”	uOR	95% CI	aOR	95% CI
			*n* (%)	*n* (%)				
Group	Control (TB−)	97 (50.5)	43 (44.3)	54 (55.7)	Ref	–	Ref	–
	Cases (TB+)	95 (49.5)	29 (30.5)	66 (69.5)	**1.8** *	**1.00–3.3**	0.7	0.2–2.7
Sex	Male	147(76.6)	59 (40.1)	88 (59.9)	Ref	–	Ref	–
	Female	45 (23.4)	13 (28.9)	32 (71.1)	1.7	0.8–3.4	2.3	0.9–5.5
Age					1	0.9–1.02	–	–
Marital Status	Married	88 (45.8)	27 (30.7)	61 (69.3)	Ref	–	–	–
	Single	34 (17.7)	16 (47.1)	18 (52.9)	0.5	0.2–1.1	–	–
	Other	70 (36.5)	29 (41.4)	42 (58.6)	0.62	0.3–1.2	–	–
Profession	Employed	97 (50.5)	43 (44.3)	54 (55.7)	Ref	–	Ref	–
	Unemployed	95 (49.5)	29 (30.5)	66 (69.5)	**1.8** *	**1.0–3.3**	0.9	0.4–1.9
BMI, kg/m^2^	<18.5	65 (33.8)	19 (29.2)	46 (70.8)	1.8	0.9–3.6	0.9	0.4–2.4
	18.5–25	84 (43.8)	36 (42.8)	48 (57.1)	Ref	–	Ref	–
	>25	43 (22.4)	17 (39.5)	26 (60.5)	1.14	0.5–2.4	1.1	0.4–2.8
Educational level	Literate	131 (68.2)	61 (46.6)	70 (53.4)	Ref	–	**Ref**	–
	Illiterate	61 (31.8)	11 (18.0)	50 (82.0)	**3.9** *	**1.9–8.3**	**2.5** *	**1.0–6.1**
Smoking	No	123 (64.1)	54 (43.9)	69 (56.1)	Ref	–	Ref	–
	Yes	69 (36.0)	18 (26.1)	51 (73.9)	**2.2** *	**1.2–4.2**	**2.4** *	**1.1–5.3**
Alcoholism	No	152 (79.2)	56 (36.8)	96 (63.2)	Ref	–	–	–
	Yes	40 (20.8)	16 (40)	24 (60)	0.9	0.4–1.8	–	–
Washing Hands before Meal	Regularly	136 (70.8)	50 (36.8)	86 (63.2)	Ref	–	–	–
	Occasionally	56 (29.2)	22 (39.3)	34 (60.7)	0.9	0.5–1.7	–	–
Handwash after Defecation	No	73 (38.0)	36 (49.3)	37 (50.7)	Ref	–	Ref	–
	Yes	119 (62.0)	36 (30.3)	83 (69.8)	**2.3** *	**1.2–4.1**	**2.4** *	**1.2–4.7**
Use of Toilets	No	2 (1.0)	0 (0)	2 (100)	–	–	–	–
	Yes	190 (99.0)	72 (37.9)	118 (62.1)	–	–	–	–
Elimination of Dirty Water	Channels	8 (4.2)	2 (25.0)	6 (75.0)	3.1	0.6–16.0	3.3	0.5–20.0
	Septic Tanks	93 (48.4)	47 (50.5)	46 (49.5)	Ref	–	Ref	–
	On the Road	91 (47.4)	23 (25.27)	68 (74.7)	**3.0** *	**1.6–5.6**	2.0	0.9–4.7
Deworming 6 Months Before	No	150 (78.1)	48 (32)	102 (68)	**2.8** *	**1.4–5.7**	1.8	0.7–4.4
	Yes	42 (21.9)	24 (57.1)	18 (42.9)	Ref	–	Ref	–
Coughing over 15 Days?	No	96 (50.0)	42 (43.8)	54 (56.3)	Ref	–	Ref	–
	Yes	96 (50.0)	30 (31.3)	66 (68.8)	1.7	0.9–3.1	1.1	0.4–3.1
Animals in the Yard	No	104 (54.2)	36 (40.9)	52 (59.1)	Ref	–	–	–
	Yes	88 (45.8)	36 (34.6)	68 (65.4)	1.3	0.7–2.4	–	–

aOR = adjusted odds ratio; BMI = body mass index; Ref = reference; uOR = unadjusted odds ratio.

* *P*-value <0.05.

The bold font indicate the results are significant.

In our study, nonliterate participants were twice as likely as literate ones to have a parasitic infection (aOR: 2.5; 95% CI: 1.01–6.1) (Table 3). This result is supported by the study of Almeida et al.,^47^ in which low educational level influenced the risk of contracting a parasitic infection (*P* <0.05). This would be a corollary of the lack of knowledge and ignorance of the modes of parasite infection, all of which suggests the need to deploy integrated health education strategies to control the disease. The study also reported that those who smoked were twice as likely to have a parasite infection compared with those who did not smoke (aOR: 2.4; 95% CI: 1.1–5.3). Similar results were reported in a study in Iran (OR: 0.3; 95% CI: 0.19–0.65; *P =* 0.001).^48^ Also in this study, those who washed their hands after defecation were twice as likely to have a parasitic infection (aOR: 2.4; 95% CI: 1.2–4.7). Similar results have been reported elsewhere in Ethiopia (OR: 3.03; 95%: CI: 1.01–5.05) and Cameroon.^49^ This fact should indicate otherwise, but we assume that there were biased reports from participants that the water used after defecation was contaminated with parasites and therefore contaminated the hands, which would sustain the transmission cycle.

### Limitations of the study.

Our study had some limitations. The participants provided only one stool or urine sample. It would have been interesting to collect three consecutive samples to increase the sensitivity and diagnostic accuracy. Also, we did not perform the Baermann method for *Strongyloides stercoralis*. Only microscopy was performed and not quantitative polymerase chain reaction (qPCR), which has a higher yield. In addition, serology was not performed, and previous studies documented that immunity does not normalize after helminth infection; therefore, the risk of previous/recent helminth coinfection on TB progression was not able to be evaluated. This was a pilot study with a small sample size; a large-scale longitudinal study is highly desirable as well as the use of qPCR for monitoring and evaluation of deworming programs.

## CONCLUSION

Despite the limitations, our study contributes significantly to existing knowledge of the coinfection of TB and parasitic infections. This study reported a high frequency of parasitic infections (protozoa and helminths) in TB patients compared with non-TB patients. Considering their mode of infection as well as the factors associated with parasitic infection encountered in our study, public health interventions are essential. These could include safe water supply, community health education, food hygiene and sanitation maintenance, and systematic parasite diagnosis in TB cases. These interventions should be integrated into TB program action plans in support of the neglected tropical diseases program.

In addition, our study reported a diversity of helminths (*S. mansoni*, *D. dendriticum*, *E. vermicularis*, *W. bancrofti,* and *H. nana*), which are believed to play a major role in host immunological disturbances, making the host susceptible to TB. Exploratory studies on helminth and mycobacteria interactions need to be conducted in our context.

## Supplemental Materials

10.4269/ajtmh.23-0346 Supplemental Materials
