# Supplementary material for: Intestinal Protozoa, Helminth Infection, and Associated Factors among Tuberculosis Patients and Nontuberculosis Persons in Bobo-Dioulasso City, Burkina Faso
Source: Am J Trop Med Hyg. 2024 Sep 24;111(6):1265–72. doi: 10.4269/ajtmh.23-0346 (PMC11619520; doi:10.4269/ajtmh.23-0346)
Supplement: Supplemental Materials [file tpmd230346.SD1.pdf]

Intestinal protozoa, helminth infection, and associated factors among tuberculosis patients and non-tuberculosis persons in Bobo-Dioulasso city, Burkina Faso.

Abstract: Tuberculosis (TB) and parasite co-infection is a public health issue in developing countries despite mass drug distribution, health promotion, and education. The current comparative cross-sectional study reported the frequency and associated factors of TB and Parasite co-infection from newly pulmonary TB patients (TB+) and non-TB participants (TB-) in Bobo-Dioulasso from 2019 to 2021. From a total of 192 participants involved 95 were TB+ and 97 non-TB.There was no statistically significant difference in parasitic infection between the two groups, although it was higher in TB+ than TB- (69.47% (66/95) *versus* 55.67% (54/97), p=0.069). Protozoa infestations were significantly higher in TB+ than in TB- (61.05% *versus* 37.11%, p=0.001). Specifically, *Entamoeba spp.* and *Cryptosporidium spp.* followed this pattern with (35.79% *versus* 19.59%, p=0.001) and (22.11% *versus* 8.25%, p=0.012), respectively. Although higher in TB+ patients, the frequency of helminthiasis was not significantly different between the two groups (23.16% *versus* 15.46%, p=0.177). Helminths species were *Schistosomia mansoni* (17.89% *versus* 12.37%), *Dicrocoelium dentriticum* (3.16% *versus* 1.03%), *Enterobius vermicularis* (2.11% *versus* 2.06%), *Wucherera bancofti* (1.1% *versus* 0.0%) and *Hymenolepis nana* (1.1% *versus* 0.0%). Illiteracy (AOR, 2.48; 95%CI:1.01-6.09), smoking (AOR, 2.39; 95%CI:1.07-5.32), and hand washing after defecation (AOR, 2.35; 95%CI:1.17-4.71) were the factors associated with Parasites. This study reported high frequency of parasite coinfection in TB patients. These findings suggest the implementation of adequate health education for behavioral change and a policy of systematically diagnosing parasites in TB patients for better management of co-infection.

Key words: Intestinal protozoa and helminth, Tuberculosis, Co-infection frequency, Associated factors, Burkina Faso
